# A personalized online intervention to enhance back pain-related self-efficacy: A two-arm randomized controlled trial (IDRIS)

**DOI:** 10.1016/j.invent.2025.100892

**Published:** 2025-11-25

**Authors:** Paul Hüsing, Mareike Busmann, Bernd Löwe, Petra Engelmann

**Affiliations:** a Department of Psychosomatic Medicine and Psychotherapy, University Medical Center Hamburg-Eppendorf, Martinistraße 52, 20251, Hamburg, Germany

**Keywords:** Low back pain, Digital intervention, Psychoeducation, Self-efficacy, Personalization

## Abstract

**Objective:**

Low back pain (LBP) affects daily functioning and strains healthcare systems. Cognitive, behavioral, and emotional factors contribute to its persistence, yet these factors are often neglected in standard care. Personalized digital interventions containing psychoeducational information on relevant biopsychosocial aspects may thus improve outcomes when applied to patients in an early stage. This trial examined whether a brief, personalized animated psychoeducational video—tailored to individual risk profiles based on patient-reported outcome measures (PROMs) and covering biopsychosocial contributors to LBP (depression, catastrophizing, health anxiety, fear of movement, pain endurance behavior, expectations, trauma, emotion regulation)—improves pain-related self-efficacy in adults with acute and subacute low back pain (0–12 weeks).

**Methods:**

In this two-arm randomized controlled trial, participants with back pain (duration <12 weeks) and resulting impairment were randomized to receive either a personalized animated video (intervention) or care as usual (control). Videos comprised modules (≈2–3.5 min per module) selected from eight possible topics based on baseline PROM cut-offs. Outcomes were assessed at baseline, 4 weeks and 12 weeks. Primary analysis used mixed ANOVA to examine changes in pain-related self-efficacy (FESS) over time and between groups; secondary outcomes on biopsychosocial factors were tested with repeated measures ANOVAs and group-adjusted ANCOVAs.

**Results:**

75 participants were included in the analysis. Pain-related self-efficacy improved significantly over time, F(2, 148) = 6.435, *p* = .002, but without significant group differences, F(2, 148) = 2.146, *p* = .121. Most secondary outcomes also improved (all *p* < .001), except pain avoidance-endurance behavior, but analyses did not yield significant differences between intervention and control group (all *p* > .05. Participants rated the intervention as credible (M = 8.36), with moderate ratings for personal fit and symptom benefit. No adverse events were reported.

**Conclusion:**

Although brief exposure may have limited the impact, the intervention was well-received. Future research should explore integrating personalized psychoeducation into multimodal treatments, emphasizing the importance of individualized approaches for this diverse patient population.

**Summary:**

A brief personalized online intervention improved back-pain self-efficacy, without being superior to an untreated control group. High acceptance seems promising for future multimodal use.

## Highlights

• This RCT tested a brief personalized online video for (sub)acute low back pain.
• Both intervention groups improved self-efficacy, without between-group differences.
• Intervention group showed psychosocial improvements over three months.
• Participants rated the intervention highly credible and moderately useful.
• Intervention is feasible, well-accepted; promising for multimodal care pathways.

## 1 Introduction

Low back pain (LBP) is a leading global health concern, affecting over 600 million people and projected to exceed 800 million by 2050 (Ferreira et al., 2023). While many acute cases resolve, a significant proportion of individuals experience persistent symptoms. LBP is now understood as a complex biopsychosocial condition, with psychological and social factors often outweighing biological ones in predicting chronicity (Balagué et al., 2012; Hartvigsen et al., 2018). Among the psychological factors in LBP, depression, anxiety, catastrophizing, pain-endurance behavior, negative symptom expectations, fear of movement and inactivity, deficits in emotion regulation, and traumatic experiences are frequently implicated (Hayden et al., 2019; Nieminen et al., 2021; Ravyts et al., 2024; Stevans et al., 2021; Thomas et al., 2024; Vlaeyen et al., 2016; Wertli et al., 2014). Yet, treatments for LBP often remain narrowly biomedical (Dionne et al., 2001; Glombiewski, 2018). At the same time and despite the strong evidence supporting cognitive, behavioral, and emotional influences on LBP, access to psychological care is often limited due to provider shortages, stigma, or lack of awareness (Scott et al., 2010; Slade et al., 2009).

“Evidence” hereby refers to longitudinal research identifying psychological risk factors that predict poor recovery from acute LBP (Foster et al., 2010; Pincus and McCracken, 2013; Wertli et al., 2014). The transition from acute to chronic low back pain has been conceptualized by mechanistic models such as the fear-avoidance model (Vlaeyen et al., 2016) and cognitive-behavioral frameworks that highlight the roles of catastrophizing, fear of movement, and negative outcome expectations in maintaining pain and disability (Balagué et al., 2012). Psychosomatic models—such as perceptual inference accounts (Van den Bergh et al., 2017) and the Persistent Physical Symptoms framework (Löwe et al., 2024) —add complementary mechanisms by emphasizing how expectations, attention and central processing shape symptom perception and persistence; together these frameworks justify targeting cognitive appraisals and expectations in early psychoeducation. Self-efficacy—patients' confidence in performing activities despite pain—has been shown to mediate the relationship between pain and disability and to predict functional outcomes (Jackson et al., 2014; Riley et al., 2020). It can be regarded as the key mechanism in the relationship between pain and disability (Costa Lda et al., 2011). Therefore, brief explanatory psychoeducation that reduces dysfunctional psychological factors while promoting self-efficacious coping is a theoretically grounded strategy to reduce the risk of chronification.

Personalized (psychological) treatment approaches are increasingly supported by meta-analytic evidence (Nye et al., 2023). Collecting individualized data through validated patient-reported outcomes (PROMS) can guide treatment, monitor symptoms, detect deterioration early, and improve patient-clinician communication (Lutz et al., 2022). For example, baseline PROMs can be used in combination with predefined, literature-derived cut-offs to provide orientation in treatment planning. Compared to clinician-led individualization, PROM-based tailoring is scalable, transparent and reproducible; compared to machine-learning or ecological-adaptive tailoring, it is simpler and less dynamic but easier to implement in low-resource settings (Pruski et al., 2025). Despite being common in somatic medicine, PROMS remain underused in psychosocial contexts. At the same time, digital interventions offer an efficient, scalable solution for delivering personalized care. Benefits include flexibility, anonymity, reduced wait times, and integration across disciplines (Buhrman et al., 2016). A meta-analysis of 36 RCTs on digital psychological interventions for chronic pain found small to moderate effects on pain intensity, impairment, self-efficacy, depression, anxiety, and catastrophizing (Gandy et al., 2022). Notably, patient acceptance of digital tools can be significantly increased through brief informational videos (Baumeister et al., 2015). Digital psychoeducation, in particular, holds promise as a low-threshold entry point to care. A Cochrane review showed that just 2.5 h of in-person psychoeducation improved back pain outcomes and return-to-work timelines in acute and subacute (≤ 3 months) back pain (Engers et al., 2008), though many interventions lacked clear theoretical grounding. This suggests digital formats—especially when personalized—could match or exceed these benefits while addressing logistical barriers.

Given the diverse needs of patients with LBP, combining biopsychosocial education with digital delivery and personalized content appears especially promising. Tailoring psychoeducational tools to individual risk profiles, symptom patterns, and expectations could enhance treatment relevance and acceptance. As digital health technologies advance, integrating PROMS and personalized feedback into digital platforms may bridge existing care gaps and improve long-term outcomes for people with back pain. In our study, we aimed to examine the effects of individualized, psychoeducational animated videos on self-efficacy and functioning in patients with LBP.

## 2 Methods

### 2.1 Study design and setting

**Fig. 1:**
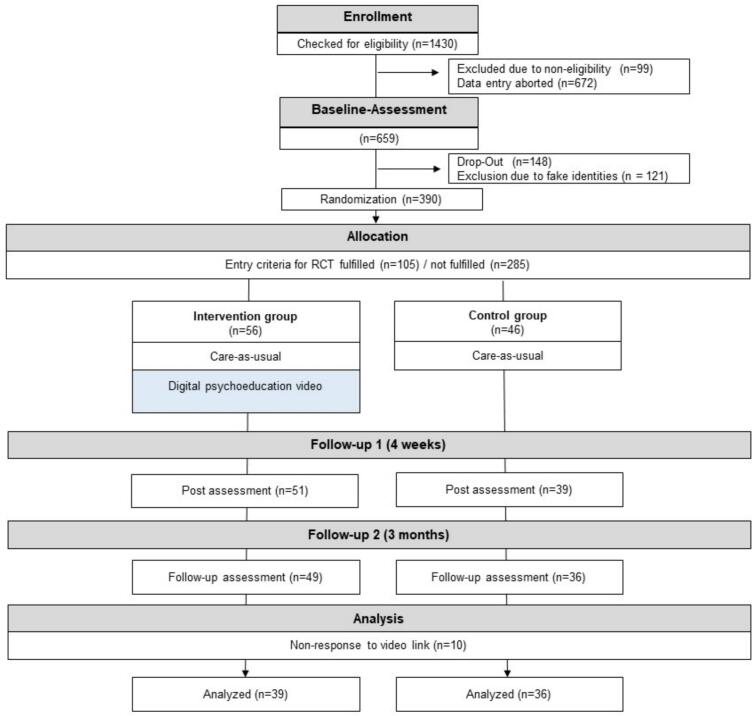
Flowchart of the IDRIS intervention study, adapted from the CONSORT statement (Hopewell et al., 2025). Patients who did not fulfill entry criteria for the RCT study (*n* = 285) were included in the overall trial analysis.

Data in this study was derived from the IDRIS trial (full title: “From the identification of biopsychosocial risk factors to an increase in pain-related self-efficacy – The online-based conveyance of an explanatory model for chronic back pain”). In this prospective online study, we used a “Cohort multiple randomized controlled trial”-Design (Relton et al., 2010) with patients suffering from acute (0–4 weeks) or subacute (4–12 weeks) LBP, resulting in one observational study sample to investigate risk factors for LBP chronification, which will be reported elsewhere, and a subsample used for the RCT described in this study (see 2.2 and Fig. 1). All patients were recruited across Germany by using social media (Instagram, Facebook) and flyers in specific back pain-related settings (e.g., physiotherapy and orthopaedic practices, pharmacies, GP offices). Assessments were exclusively conducted online at baseline (T0), after four weeks (T1) and after twelve weeks (T2). Ethical approval was granted by the Local Psychological Ethics Committee (LPEK) at the Center for Psychosocial Medicine of the University Medical Centre Hamburg-Eppendorf on December 9, 2021 (LPEK-0393). The study has been prospectively registered at the German Clinical Trials Register (Deutsches Register Klinischer Studien, 2021) (DRKS00025445) and the corresponding study protocol has been published (Engelmann et al., 2022). The study was conducted and reported in accordance with the Strengthening the Consolidated Standards of Reporting Trials (CONSORT) guidelines (Hopewell et al., 2025), with the corresponding check-list included in Appendix 1.

### 2.2 Study hypotheses

Our study aims were to assess the impact of video-animated psychoeducation on pain-related self-efficacy in patients with LBP. We developed animated videos to help patients understand contributing factors to their back pain and to demonstrate behavioral, cognitive, and emotional strategies for improvement. We hypothesized that this intervention would lead to increased pain-related self-efficacy compared to an untreated control group that did not receive this information (I). Additionally, we aimed to explore the effects of personalized psychoeducation on functional outcomes and biopsychosocial risk factors for chronic LBP (II). Thirdly, we evaluated acceptance and fit of the intervention to individual patients' needs (III).

### 2.3 Study participants

Patients were able to participate in the study if they were aged between 18 and 67 years, suffered from back pain for no longer than 12 weeks, provided contact details, and gave online informed consent. There was no minimum pain duration. Exclusion criteria were chronic back pain (duration >12 weeks), a severe acute somatic back injury (herniated disc, surgical intervention, etc.), insufficient knowledge of German, a self-reported substance use disorder, and insufficient self-reported mental or physical health for study participation. Insufficient self-reported mental or physical health referred to self-reported inability to participate in an online study (e.g., acute severe somatic injury, inability to use the online survey). For inclusion in the randomized subsample, participants additionally had to meet threshold scores indicating a minimum psychological (Mental Composite Scale (MCS-12) score of ≤50 in the Short Form 12 Health Survey (SF-12) (Ware Jr. et al., 1996)) and somatic symptom burden (mean numeric rating scale score (NRS) ≥ 5 in two recommended numerical rating scales (Rief et al., 2017)).

Explicit exclusion criteria for participation in the RCT cohort were a current or previous inpatient psychosomatic hospital or rehabilitation treatment, and a current or previous outpatient psychotherapy with a focus on pain.

### 2.4 Procedures

Screening and eligibility assessment were fully digital via the web platform REDCap (Research Electronic Data Capture) (Harris et al., 2009). REDCap is a secure, web-based software platform designed to support data capture for research studies. Research staff performed automated checks and manual identity verification where suspicious entries were detected; no clinician-administered diagnostic screening was performed prior to randomization. General inclusion criteria for the study were checked after participants clicked on a link provided online or on print-outs. After providing informed consent as well as contact details and inclusion in the study, participants received a link to the online survey. Study data was also assessed on REDCap. Eligible participants for the RCT were automatically included in the RCT cohort at baseline and randomly assigned to either the intervention or the control group. Randomization took place directly after inclusion, based on a random number system and a web-based randomization tool. Due to the mandatory nature of the digital assessment tool, no missing values were recorded in the dataset.

### 2.5 Study arms

Only participants randomized in the intervention group received a video intervention after baseline assessment. Participants randomized to the control group received care as usual (no video). The non-RCT population consisted of cohort participants who were followed observationally (they either did not meet RCT threshold criteria or were not randomly selected for RCT participation) and completed the same follow-up questionnaires as the intervention group after four and twelve weeks. Data on the non-RCT population is described elsewhere (Engelmann et al., 2025).

#### 2.5.1 Intervention

The intervention consisted of an individually tailored animation video covering different risk factors for chronification of back pain. Out of a total number of eight different modules, parts were included if corresponding cut-off scores were fulfilled at baseline assessment (see Table 1). Participants received a personal link to their individual video and could access the content for a total duration of seven days. After three days, a reminder e-mail was sent out. Modules on risk factors were selected based on literature research and guidelines on chronic back pain (Engelmann et al., 2022). Cut-offs were derived from publications on validation and clinical implementation of the instruments used for assessment of the modules.

**Table 1:** Risk factors, PROMs and cut-off scores used to personalize the animated video intervention.

Risk factor	Instrument (PROM)	Cut-off score	Approx. video length	Module content summary
Depression severity	PHQ-8	≥5	≈ 02:55 Minutes	Describes the bidirectional relationship between low mood and back pain, emphasizing behavioral and cognitive vicious cycles. Highlights the importance of early help-seeking, social support, and re-engagement in pleasurable and active daily routines to improve both mood and pain outcomes.
Health anxiety	WI-7	≥2	≈ 03:11 Minutes	Explains how fear and hypervigilance toward bodily sensations can amplify pain perception through autonomic arousal and muscle tension. Promotes relaxation, breathing exercises, and open communication about fears as effective ways to reduce anxiety-related pain amplification.
Catastrophizing	CSQ-CAT	≥10	≈ 02:42 Minutes	Explains how catastrophic thinking (exaggerating pain consequences, feeling helpless) increases fear and avoidance, sustaining pain. Illustrates how cognitive reappraisal and attention diversion can reduce catastrophizing and promote adaptive coping. Encourages self-practice and, if needed, professional support.
Pain endurance	AE-FS	PPS subscale ≥3	≈ 03:09 Minutes	Differentiates between avoidance and overexertion coping styles. Promotes balanced pacing and gradual activity increase to prevent both inactivity-related deconditioning and overuse injuries, fostering sustainable movement habits and resilience.
Fear of movement and physical inactivity	TSK-GV and IPAQ-SF	≥ 25	≈ 02:42 Minutes	Addresses kinesiophobia and the “vicious cycle of rest and pain.” Encourages gradual exposure and stepwise reactivation to rebuild confidence in movement, improve physical conditioning, and harness the body's natural pain-inhibition mechanisms.
		≤ Moderate physical activity		
Symptom and treatment expectations	TEX-Q and NRS (EXPECT 1,2,3)	Positive expectations ≤6 & Negative expectations ≥5	≈ 03:07 Minutes	Describes how positive and negative expectations shape pain perception via placebo and nocebo mechanisms. Encourages realistic, optimistic thinking and re-evaluation of negative expectations, highlighting that focusing on positive experiences can improve recovery trajectories.
		Mean score ≥ 5		
Traumatic experiences	ITQ	≥13	≈ 02:48 Minutes	Explains links between early or recent traumatic experiences, stress regulation, and chronic pain vulnerability. Encourages seeking professional support to process trauma, regain emotional stability, and improve pain management through psychotherapy and guided care.
Emotion regulation deficits	ERQ	Reappraisal ≤4.0	≈ 03:30 Minutes	Illustrates how difficulties in identifying and managing emotions can intensify pain. Encourages emotional awareness and labeling, use of emotion diaries, and constructive expression of feelings to support healthier coping and reduce stress-related pain responses.
		& Suppression ≥3.5		

Note. PHQ-8 = Patient Health Questionnaire-8, WI-7 = Whiteley-Index Short Version, CSQ-CAT = Coping Strategies Questionnaire - Subscale, AE-FS = Avoidance-Endurance Fast Screening, PPS = Pain Persistence Subscale, TSK-GV = Tampa Scale for Kinesiophobia, IPAQ-SF = International Physical Activity Questionnaire, TEX-Q = Treatment Expectation Questionnaire, NRS = Numeric Rating Scale, ITQ = International Trauma Questionnaire, ERQ = Emotion Regulation Questionnaire.

For each module, a video sequence of approximately the same length was produced using Adobe After Effects (Christiansen, 2013). Before animation, text scripts with information from guidelines and treatment recommendations were produced (Engelmann et al., 2022). The content integrates cognitive–behavioral principles (targeting catastrophizing and fear-avoidance; (Vlaeyen and Linton, 2012)), Bandura's self-efficacy framework (promoting mastery and graded activity; (Bandura, 1977)), and expectation/perception models from psychosomatic research (addressing symptom interpretation; (Löwe et al., 2024)).

The scripts were written in lay language and used evidence-based feedback strategies such as the use of examples and metaphors, validation and prompting strategies (Brehaut et al., 2016). The scripts were voice recorded and visualized using stock footage (full scripts and videos are included in the e-appendix). In the English version of the videos, an AI-assisted text-to-speech-software was used for voice recordings (ElevenLabs, 2025). Prior to the trial, videos were pilot-tested with a small sample (*n* = 4) of trained clinicians for comprehensibility and technical functioning.

### 2.6 Assessment and outcomes

We collected comprehensive sociodemographic variables to characterise the cohort, enable potential subgroup and covariate analyses, and assess external validity of findings. Additional validated questionnaires were used at all three times of assessment. A complete overview on all data and time points of respective data collection can be found in the study protocol (Engelmann et al., 2022).

#### 2.6.1 Primary outcome

The primary outcome of this study was change in patients' pain-specific self-efficacy after 1 month, determined via the German version of the Pain Self-Efficacy Questionnaire (“Fragebogen zur Erfassung der schmerzspezifischen Selbstwirksamkeit”; FESS) (Mangels et al., 2009), a German adaptation of the original instrument (PSEQ) developed by Nicholas (Nicholas, 2007). Its sound psychometric properties, including construct and convergent validity, predictive value, and internal consistency, have been demonstrated in a sample of 363 chronic pain patients in an orthopaedic rehabilitation hospital (Mangels et al., 2009).

#### 2.6.2 Secondary outcomes

Secondary outcomes were changes in symptom intensity and symptom impairment after 1 and 3 months, assessed using two numerical rating scales according to the recommendations of the EURONET-SOMA network (Rief et al., 2017), along with the biopsychosocial risk factors summarized in Table 1: namely, depression severity (Patient Health Questionnaire-8) (Löwe et al., 2004), health anxiety (Whiteley-Index short version) (Pilowsky, 1967), catastrophizing (Coping Strategies Questionnaire – Subscale) (Rosenstiel and Keefe, 1983), pain endurance (Avoidance-Endurance Fast Screening) (Wolff et al., 2020), fear of movement (Tampa Scale for Kinesiophobia) (Miller et al., 1991) and inactivity (International Physical Activity Questionnaire) (Craig et al., 2003), symptom and treatment expectations (Treatment Expectation Questionnaire) (Shedden-Mora et al., 2023), traumatic experiences (International Trauma Questionnaire) (Cloitre et al., 2018), and emotion regulation deficits (Emotion Regulation Questionnaire) (Gross and John, 2003). Perceived usefulness of the intervention and personal fit were measured using a NRS (1−10), similar to previous studies on explanatory models (Weigel et al., 2023).

### 2.7 Statistical analyses

Internal consistency of each psychometric scale was assessed using Cronbach's alpha, with values above 0.70 considered acceptable. Differences at baseline between the intervention and control group were analyzed using independent samples *t*-tests or chi-square tests. Analyses were performed as prespecified in the study protocol (Engelmann et al., 2022). The primary hypothesis (group × time interaction for self-efficacy) was tested using a two-way mixed ANOVA. ANCOVAs were conducted for planned adjusted comparisons. Given the small and balanced sample with minimal missing data, the mixed ANOVA was considered appropriate and consistent with the a priori analysis plan. For the second hypothesis (the psychoeducational intervention reduces functional outcomes and biopsychosocial risk factors), changes in symptom intensity, symptom interference and biopsychosocial risk factors over time were analyzed using repeated measures ANOVA. Risk factors included depressive symptoms, health anxiety, catastrophizing thoughts, pain endurance behavior, fear of movement and physical inactivity, and symptom and treatment expectations. Due to the design of the study, traumatic experiences and emotion regulation deficits were only assessed at baseline and thus not included in the analysis. To examine differences between intervention and control group, an ANCOVA with experimental group (personalized psychoeducation vs. no psychoeducation) as independent variable and changes in functional impairment and biopsychosocial risk factors from baseline to 3-month follow-up as dependent variables was used, with covariates age and gender. For the third hypothesis (acceptance and fit of the intervention), we analyzed data descriptively. Results were reported using regression coefficients, corresponding 95-%-confidence intervals and *p*-values. Statistical analyses were performed using IMB SPSS Version 27 (IBM Corp, 2020). Due to forced entry method, no missing values were expected. In case participants stopped entry, up to three reminders to complete the questionnaires were send out via e-mail. If patients did not respond, they were excluded from the study.

#### 2.7.1 Sample size calculation

The study intervention is a brief intervention in the initial stage of symptoms in untreated patients, for which we assumed a small effect f = 0.15 (Cohen, 2013). We further assumed a correlation of *r* = 0.5 of the primary outcome of change in self-efficacy experience (∆ FASS) with fear of movement (∆ TSK-GV). Fear of movement was included as a covariate in the design and enabled an increase in the expected effect to f = 0.173 (Lipsey, 1990). In order to be able to demonstrate this effect size with an analysis of covariance with two groups (F-test), a covariate with α = 0.05 and power = 1-β = 0.8, a case number of *n* = 132 in both the intervention and control group was required. The number of cases was calculated using G*Power (Faul et al., 2009).

## 3 Results

Patients were recruited between December 19, 2022, and April 13, 2024. Patient recruitment and allocation are summarized in the study flowchart (Fig. 1). Of 1430 online registrants, 659 completed the eligibility questionnaire and qualified for participation. Of these, 269 were excluded for missing baseline assessments or using fictitious identities. Among the remaining 390, 105 met criteria for RCT enrollment, while 285 were included only in the overall trial (described elsewhere). After randomization, 56 participants were assigned to the intervention group and 46 to the control group. Follow-up losses totaled 7 in the intervention group and 10 in the control group. Additionally, 10 intervention participants did not respond to the video link and were excluded from analyses.

Baseline characteristics of the sample are summarized in Table 2. No significant differences could be found between the intervention and control group at baseline (all *p* > .05). Internal consistency for all measures was within acceptable or excellent range, except for the CSQ catastrophizing subscale (Cronbach's α = 0.67).

**Table 2:** Patient baseline characteristics (*N* = 75).

		Intervention group (*n* = 39)			Control group (*n* = 36)		
Variable	Instrument			Inclusion in animated video, n (%)			t or χ^2^; p-value
Sociodemographics							
Age, M (SD, range)		39.13	(11.69)		37.33	(11.11)	0.68, *p* = .50
Gender, n (%)							0.57; *p* = .45
female		31	(79,5 %)		31	(86.1 %)	
Male		8	(20.5 %)		5	(13.9 %)	
diverse		0	0		0	0	
BMI M (SD)		24.75	(4.121)		25.78	(7.23)	−0.76, p = .45
Education, n (%) higher level		16	(41 %)		22	(61.1 %)	3.02, *p* = .08
Born in Germany, n (%)		33	(84.6 %)		32	(88.9 %)	0.30, *p* = .59
Employment status, n (%) currently employed		28	(71.8 %)		25	(69.4 %)	0.002, *p* = .97
Relationship status, n (%) currently in a partnership		26	(66.7 %)		23	(63.9 %)	0.06, *p* = .80
Risk factors at baseline							
Depression severity (M, SD)	PHQ-8	15.36	(6.01)	38 (97.4 %)	14.06	(5.08)	1.01, *p* = .32
Health anxiety (M, SD)	WI-7	4.59	(2.33)	33 (84.6 %)	4.69	(1.82)	−0.22, *p* = .83
Catastrophizing (M, SD)	CSQ-CAT	7.33	(2.83)	37 (94.9 %)	7.72	(2.37)	−0.64, *p* = .52
Pain endurance (M, SD)	AE-FS	2.45	(1.32)	14 (35.9 %)	2–88	(1.13)	−1.51, *p* = .14
Fear of movement (M, SD)	TSK-GV	29.33	(8.93)	23 (59 %)	27.92	(8.04)	0.72, *p* = .47
Symptom and treatment expectations (M, SD)	TEX-Q	6.04	(1.67)	8 (20.5 %)	5.92	(1.41)	0.35, *p* = .73
	NRS EXPECT	5.49	(1.2)		5.69	(0.92)	−0.83, *p* = .41
Traumatic experiences (M, SD)	ITQ	24.1	(13.53)	28 (71.8 %)	21.11	(14.02)	0.94, *p* = .35
Emotion regulation deficits (M, SD)	ERQ	3.96	(1.21)	8(20.5 %)	3.94	(0.95)	0.1, *p* = .46
Back pain at baseline							
Back pain intensity past 7 days (M, SD)	NRS	7.62	(1.48)		7.69	(1.06)	−0.26, p = .79
Back pain impairment past 7 days (M, SD)	NRS	7.38	(1.71)		7.11	(1.82)	0.671, p = .50
Pain-related self-efficacy (M, SD)	FESS	30.1	(8.46)		33.31	(11.14)	−1.41, *p* = .16
Back pain duration, weeks (M, SD)		4.62	(4.17)		3.83	(3.86)	0.84, p = .41

*Note.* Higher level education = German *Abitur* or higher; PHQ-8 = Patient Health Questionnaire-8; WI-7 = Whiteley-Index Short Version; CSQ-CAT = Coping Strategies Questionnaire – Subscale; AE-FS = Avoidance-Endurance Fast Screening; TSK-GV = Tampa Scale for Kinesiophobia; IPAQ-SF = International Physical Activity Questionnaire; TEX-Q = Treatment Expectation Questionnaire; NRS = Numeric Rating Scale; ITQ = International Trauma Questionnaire; ERQ = Emotion Regulation Questionnaire; FESS = Questionnaire on pain-related self-efficacy.

### 3.1 Hypothesis 1: increase in pain-related self-efficacy

**Fig. 2:**
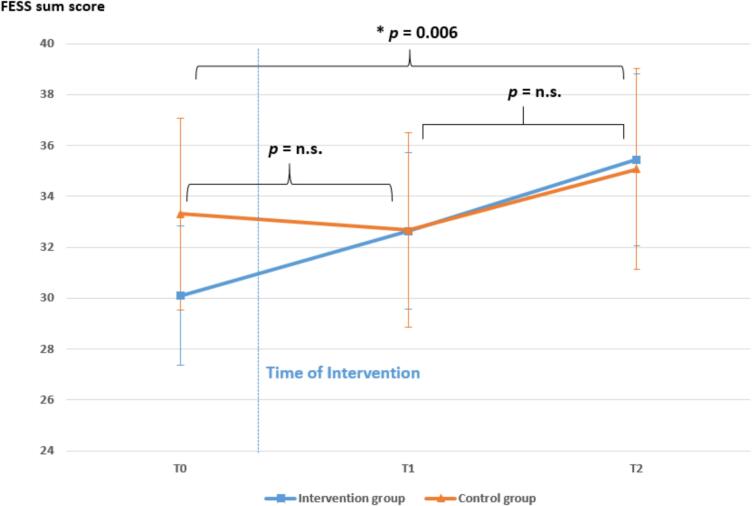
Changes in FESS scores from baseline to three-month follow-up (T2), *p*-values correspond to change over time across both groups. Error bars indicate 95 % confidence intervals.

Regarding our first hypothesis, after adjusting for age, sex and fear of movement, no statistically significant difference in change of pain-related self-efficacy from baseline to one-month follow-up was found between the two groups, *F* (4, 70) = 2.293, *p* = .134. We did only find a statistically significant overall improvement in self-efficacy (FESS) over time across both groups, *F* (2, 148) = 6.435, *p* = .002, partial η^2^ = 0.08 (Fig. 2). Bonferroni-adjusted post-hoc analysis revealed significantly (*p* = .006) higher FESS scores at the three-month follow-up compared to baseline (*M*_Diff_ = −3.57, 95 %-CI [−6.31, −0.84]), with non-significant differences between baseline and one-month follow-up (*p* = .792) and one-month and three-month follow-up (*p* = .052).

### 3.2 Hypothesis 2: changes in secondary outcomes

We first analyzed changes in secondary outcomes over time for intervention and control group separately. All secondary outcomes improved significantly in the intervention group from baseline to three-month follow-up, except for pain avoidance behavior (*p* = .673; Table 3). In the control group, however, only back pain intensity and impairment over the past 7 days improved significantly (*p* < .001), while none of the changes from T0 to T2 in the biopsychosocial risk factors reached statistical significance (all *p* > .05).

**Table 3:** Changes in secondary outcomes from baseline to 1-month follow-up (T1) to 3-month follow-up (T2) in the intervention and control group. F- and p-values refer to repeated measures ANOVA.

	Intervention group (n = 39)			F & p-values, partial η^2^	Control group (n = 36)			F & p-values, partial η^2^
Variable	T0	T1	T2		T0	T1	T2	
*Functional outcomes*								
Back pain intensity past 7 days (NRS)	7.62 (1.48)	6.59 (2.34)	5.59 (2.59)	*F*(2, 76) = 22.582, ***p* <** .**001, partial η**^**2**^ **= 0**.**373**	7.69 (1.069)	6.44 (2.48)	6.0 (2.38)	*F*(2, 70) = 12.175, ***p* <** .**001, partial η**^**2**^ **= 0**.**258**
Back pain impairment past 7 days (NRS)	7.38 (1.71)	6.41 (2.55)	5.36 (2.83)	*F*(2, 76) = 17.488, ***p* <** .**001, partial η**^**2**^ **= 0**.**315**	7.11 (1.82)	6.25 (2.74)	5.61 (2.6)	*F*(2, 70) = 10.112, ***p* <** .**001, partial η**^**2**^ **= 0**.**224**

*Risk factors*								
Depression severity (PHQ-8)	15.36 (6.01)	13.51 (5.88)	12.0 (6.05)	*F*(2, 76) = 10.399, ***p* <** .**001, partial η**^**2**^ **= 0**.**215**	14.06 (5.08)	12.92 (5.45)	12.69 (5.86)	*F*(2, 70) = 1.441, *p* = .244
Health anxiety (WI-7)	4.59 (2.33)	4.26 (2.49)	3.61 (2.56)	*F*(2, 76) = 6.636, ***p* =** .**002, partial η**^**2**^ **= 0**.**149**	4.69 (1.82)	4.39 (2.06)	3.92 (2.44)	*F*(2, 70) = 2.772, *p* = .069
Catastrophizing (CSQ-CAT)	7.33 (2.83)	6.79 (2.81)	5.71 (2.98)	*F*(2, 76) = 11.23, ***p* <** .**001, partial η**^**2**^ **= 0**.**228**	7.73 (2.37)	7.58 (2–57)	6.86 (3.02)	*F*(2, 70) = 2.876, *p* = .063
Fear avoidance behavior (AEFS)	2.45 (1.32)	2.30 (1.32)	2.31 (1.44)	*F*(1.68, 63.839) = 0.398, *p* = .673	2.88 (1.13)	2.61 (1.28)	2.58 (1.44)	*F*(2, 70) = 1.404, *p* = .252
Fear of movement (TSK-GV)	29.33 (8.93)	28.21 (9.72)	25.64 (8.77)	*F*(2, 76) = 11.022, ***p* <** .**001, partial η**^**2**^ **= 0**.**225**	27.92 (8.04)	26.97 (8.74)	26.56 (8.84)	*F*(2, 70) = 0.87, *p* = .423
Treatment expectations (TEX-Q)	6.04 (1.67)	6.27 (1.46)	6.92 (1.27)	*F*(1.686, 64.058) = 7.376, ***p* =** .**001, partial η**^**2**^ **= 0**.**163**	5.92 (1.41)	6.06 (1.74)	6.3 (1.67)	*F*(2, 70) = 1.093, *p* = .341

Significant p-values (p < .05) are indicated in bold

In the second step, we compared change scores (sum score T2 – sum score T0) between intervention and control group. However, analyses did not yield significant differences (all p > .05; Table 4).

**Table 4:** Results of ANCOVA group comparisons of change scores from baseline to three-month follow-up between the intervention and control group for secondary outcomes.

Change score from T0 to T2	Intervention group (M, SD)	Control group (M, SD)	ANCOVA group differences
∆ Back pain intensity (NRS)	−2.03 (2.17)	−1.69 (2.23)	F(1,71) = 0.55, *p* = .461
∆ Back pain impairment (NRS)	−2.03 (2.49)	−1.5 (1.95)	F(1,71) = 1.161, *p* = .285
∆ Depression severity (PHQ-8)	−3.36 (4.7)	−1.36 (5.9)	F(1,71) = 3.315, *p* = .073
∆ Health anxiety (WI-7)	−0.97 (1.87)	−0.78 (2.34)	F(1,71) = 0.258, *p* = .613
∆ Catastrophizing (CSQ-CAT)	−1.62 (2.15)	−0.86 (2.62)	F(1,71) = 1.834, *p* = .18
∆ Fear avoidance behavior (AEFS)	−0.14 (1.29)	−0.29 (1.21)	F(1,71) = 0.239, *p* = .63
∆ Fear of movement (TSK-GV)	−3.69 (5.41)	−1.36 (7.33)	F(1,71) = 2.66, *p* = .11
∆ Treatment expectations (TEX-Q)	−0.88 (1.75)	0.38 (1.63)	F(1,71) = 1.57, *p* = .21

### 3.3 Hypothesis 3: acceptance and fit of the intervention

In the 39 individually designed animated videos, the modules on depression (38 times) and catastrophizing (37 times) were included most often (see Table 1). As for the other modules, baseline cut-off scores for inclusion were met for health anxiety in 32 of the cases, followed by traumatic experiences (28 times). Fear of movement was included 23 times and pain endurance 14 times, while symptom and treatment expectations as well as emotion regulation deficits (both 8 times) were included the least often. Overall, patients rated the intervention highly credible (M = 8.36, SD = 1.56). Usefulness (M = 5.82, SD = 2.22) and their previous knowledge of the information covered in the videos as mediocre (M = 5.79, SD = 2.59), fit of the information for the individual complaints (M = 5.77, SD = 2.05) and influence on the course of treatment (M = 5.56, SD = 2.21) were rated adequate.

## 4 Discussion

This two-armed randomized controlled trial investigated the effectiveness and acceptance of a brief, personalized online psychoeducational intervention aimed at enhancing pain-related self-efficacy in individuals with (sub)acute back pain. The study found a significant improvement in pain-related self-efficacy over time across both the intervention and control group, reflecting the typical recovery pattern in acute and subacute back pain. While no significant between-group differences emerged for the primary and secondary outcomes, the intervention group showed favorable within-group trends in psychosocial measures like depression severity and fear of movement. Participants rated the digital intervention as credible and moderately beneficial, highlighting its acceptability and offering insights into the potential and limitations of brief, personalized digital interventions in routine care.

The absence of significant group differences, despite notable within-group improvements, may partly be explained by the natural course of (sub)acute back pain, where symptom resolution and psychological adjustment often occur independently of active interventions. Previous research suggests that most acute low back pain episodes improve within weeks, regardless of treatment, complicating the detection of additional effects from brief interventions (Balagué et al., 2012; Hartvigsen et al., 2018). This finding reinforces the importance of selecting appropriate patient populations and timing when evaluating brief psychosocial interventions. Furthermore, while the intervention's personalization based on individual risk profiles represented an innovative aspect, its asynchronous and entirely digital nature may have limited the depth of therapeutic engagement and impact. The intervention lacked interactive components or personal contact, elements increasingly recognized as facilitators of digital health outcomes (Baumeister et al., 2015; Buhrman et al., 2016). Even brief personal interactions, such as a single telephone consultation, have been shown to enhance patient engagement, clarify content, and foster a therapeutic relationship, ultimately strengthening treatment effects (Gandy et al., 2022). Incorporating such elements into future versions of this intervention could represent a practical and low-resource means of increasing its effectiveness.

Compared with prior subacute LBP psychoeducation trials that reported modest effects (Engers et al., 2008), our results align with a pattern of limited added benefit over usual care in heterogeneous, recovering populations. Catastrophizing has been consistently identified as a predictor for chronicity; however, our catastrophizing measure demonstrated borderline internal consistency (α = 0.67), which may have reduced sensitivity to detect between-group effects and affected module selection. We therefore recommend future trials use instruments with stronger psychometric properties for both tailoring and outcome assessment.

Treatment expectations and intervention credibility likely played a moderating role in the observed outcomes. While participants rated the intervention highly credible, personal fit and usefulness received only moderate scores. However, these results are in line with the results of a study using digital explanatory models for persistent somatic symptoms (Weigel et al., 2023). Literature on placebo and nocebo effects in chronic pain consistently highlights the powerful influence of expectations on treatment outcomes, including in digital settings (Ashar et al., 2022; Evers et al., 2018). It is possible that moderate expectation ratings and variable personal fit limited the intervention's ability to leverage expectancy effects, which could have otherwise augmented the intervention's impact. Future studies should explore more dynamic and tailored strategies for managing expectations within digital health interventions, such as adaptive messaging or interactive feedback components.

The study's findings also mirror prior research indicating modest effects of digital psychological interventions for chronic pain. A recent meta-analysis by Gandy et al. (2022) reported small to moderate improvements in pain, psychological distress, and self-efficacy following internet-based interventions. Notably, interventions with greater therapist contact or interactive features tended to yield stronger outcomes, underscoring the limitations of purely self-guided formats. The personalized video-based approach in this study may represent a useful first step in engaging patients early in the course of back pain, yet its isolated application may be insufficient to produce durable, clinically meaningful improvements.

### 4.1 Limitations

Several limitations must be acknowledged. The study's primary limitation is the recruitment shortfall, resulting in a substantially smaller sample size than originally planned. The initial power calculation required 132 participants per group to detect a small-to-moderate effect, yet only 75 participants overall were ultimately randomized. This significant under-recruitment increases the likelihood of type II errors, particularly for between-group analyses, and limits the generalizability of findings. During the data collection phase, we observed a period marked by a substantial influx of fraudulent or fabricated identities attempting to enroll in our study. This anomaly necessitated additional screening measures to preserve the integrity and validity of our participant sample, i.e. a brief stop of recruitment and double-check of identities, leading to a substantial number of excluded cases (*n* = 121, see Fig. 1). Recruitment challenges are common in digital health trials and highlight the need for diversified, sustained, and perhaps incentivized recruitment strategies in future studies.

Secondly, our analyses included only participants who completed baseline assessments and engaged with follow-ups, which may have introduced selection bias. Excluded registrants and those lost prior to baseline may differ systematically (e.g., lower digital literacy, greater socioeconomic barriers), potentially limiting generalizability and biasing effect estimates toward more engaged individuals.

A third, albeit minor, limitation involves the internal consistency of the catastrophizing subscale (CSQ-CAT), which fell slightly below conventional acceptability thresholds (Cronbach's α = 0.67). While adequate for exploratory purposes, this reduced reliability may have diminished the scale's sensitivity to detect changes over time and between groups.

Lastly, while the intervention was rated as credible, the moderate scores for personal fit and symptom benefit suggest room for improving the personalization algorithm or tailoring content delivery to enhance relevance and resonance for participants. The PROM-based cut-off tailoring assumes that baseline thresholds validly indicate which domains should be targeted; this static approach may miss contextual factors or recent fluctuations in symptoms. Moreover, relying solely on PROM thresholds assumes that module inclusion maps directly to modifiable mechanisms—an assumption that requires empirical validation. These limitations constrain immediate clinical implementation and argue for piloting blended or adaptive personalization methods in routine care.

Future iterations could consider more sophisticated personalization techniques, such as machine learning-based tailoring or ecological momentary assessments to dynamically adjust intervention content.

### 4.2 Clinical implications

Despite these limitations, the study provides valuable insights into the feasibility and acceptability of personalized, brief digital psychoeducation for (sub)acute back pain. High credibility ratings and satisfactory engagement metrics indicate that patients are receptive to receiving personalized psychoeducational content via digital formats. As healthcare systems increasingly adopt blended and digital-first care models, interventions like the one evaluated here could serve as accessible, low-threshold entry points within stepped-care approaches for back pain management.

Furthermore, the intervention's personalization framework, based on PROM-derived risk profiles, offers a replicable and scalable strategy for tailoring digital content. While brief interventions alone may offer limited standalone benefits in the context of natural recovery, their integration into multimodal treatment pathways – alongside physiotherapy, medication management, and psychological support – could enhance overall care quality and patient outcomes. The ability to deliver personalized, evidence-based psychoeducation early in the back pain trajectory may help reduce care delays, improve patient knowledge, and mitigate progression to chronicity.

Future research should build upon the findings of this study by addressing several key areas. First, larger, adequately powered trials are needed to robustly evaluate the clinical effectiveness of personalized digital interventions for back pain, particularly among higher-risk subgroups with elevated psychological distress or poor prognostic profiles. Stratified analyses based on baseline psychological burden, pain severity, or pain duration could clarify which patients are most likely to benefit from such interventions. Second, incorporating blended intervention formats that combine digital content with brief personal contacts, whether via telephone, video consultation, or asynchronous messaging, represents a promising avenue for enhancing treatment effects. Evidence suggests that even minimal therapist contact can substantially improve engagement and outcomes in digital interventions (Buhrman et al., 2016; Gandy et al., 2022). Pragmatic trials comparing purely digital versus blended models would offer valuable insights for clinical implementation. Third, future studies should investigate the role of treatment expectations and credibility as potential mediators or moderators of intervention effects. Including measures of expectations at multiple time points, alongside strategies to actively shape and manage these expectations, could optimize digital intervention outcomes. Lastly, expanding personalization techniques beyond baseline PROM-derived risk factors to include dynamic assessments (e.g., ecological momentary assessment of pain, mood, and activity) or adaptive content algorithms may further improve intervention relevance and efficacy. Advances in digital health technology and artificial intelligence offer increasing opportunities to develop truly adaptive, patient-centered interventions capable of responding to individual needs in real-time.

### 4.3 Conclusion

In conclusion, this study demonstrated that a brief, personalized online psychoeducational intervention was feasible, well accepted, and associated with meaningful within-group improvements in psychosocial risk factors for (sub)acute LBP. However, no significant differences compared to usual care were detected, likely reflecting natural recovery patterns, limited intervention intensity and restricted personalization, and underpowered analyses. Future research should focus on integrating brief digital psychoeducation within multimodal care pathways, augmenting digital interventions with personal contact, and targeting higher-risk patient subgroups. These refinements may ultimately enhance the clinical utility and impact of digital health interventions for LBP.

## Ethical approval

Ethical approval for this study was granted by the Local Psychological Ethics Committee at the Center for Psychosocial Medicine of the University Medical Center Hamburg-Eppendorf (LPEK-0393). All participants provided informed consent.

## Declaration of Generative AI and AI-assisted technologies in the writing process

During the preparation of this work, the authors used ChatGPT for spellchecking and to improve grammar as well as ElevenLabs Text to Speech to generate English voice-overs in the animated videos. After using these tools, the authors reviewed and edited the content as needed and take full responsibility for the content of the publication.

## Funding

This research received funding by the *Stiftung Psychosomatik der Wirbelsäulenerkrankungen*.

## Declaration of competing interest

PH reports research funding from the Stiftung Psychosomatik der Wirbelsäulenerkrankungen (payment made to the institution), royalties for a book chapter on somatic symptom disorder by Thieme Verlag, a scholarship for attending the ICPM 2024 conference from Carus Stiftung, he operates in his own practice for psychotherapy and receives payments by the German statutory health insurances and private health insurances. MB reports no research funding (no personal honoraria). BL reports research funding (no personal honoraria) from the German Research Foundation, the German Federal Ministry of Education and Research, the German Innovation Committee at the Joint Federal Committee, the European Commission's Horizon 2020 Framework Programme, the European Joint Programme for Rare Diseases (EJP), the Federal Ministry of Health, Germany, and the Foundation Psychosomatics of Spinal Diseases, Stuttgart, Germany. He received remunerations for several scientific book articles from various book publishers and as a committee member from Aarhus University, Denmark. He received travel expenses from the European Association of Psychosomatic Medicine (EAPM), and accommodation and meals from the Societatea de Medicina Biopsyhosociala, Romania. He received a travel grant for a lecture on the occasion of the presentation of the Alison Creed Award at the EAPM Conference in Lausanne, June 2024. He received remuneration and travel expenses for lectures at the Lindauer Psychotherapiewochen, April 2024, and the University Hospital Zurich, Department of Consultation-Liaison Psychiatry and Psychosomatic Medicine, March 2025. He is President of the German College of Psychosomatic Medicine (DKPM) (unpaid) since March 2024 and was a member of the Board of the European Association of Psychosomatic Medicine (EAPM) (unpaid) until 2022. He was a member of the EIFFEL Study Oversight Committee until 2025 (unpaid). PE reports funding from the Deutsche Forschungsgemeinschaft (DFG, German Research Foundation; GZ: EN 1376/1-1).

## Data Availability

Data and materials used in this study are available upon request from the corresponding author.

## Appendix A Supplementary data

Supplementary material 1 Image 1

Supplementary material 2 Image 2
