# Supplementary material for: A personalized online intervention to enhance back pain-related self-efficacy: A two-arm randomized controlled trial (IDRIS)
Source: Internet Interv. 2025 Nov 25;42:100892. doi: 10.1016/j.invent.2025.100892 (PMC12704078; doi:10.1016/j.invent.2025.100892)
Supplement: Supplementary file 1 — Supplementary material 1 [file mmc1.docx]

**Script for the module on Catastrophizing**

When we experience back pain for the first time, many of us automatically trigger warning signals in the form of certain thoughts and feelings: “Where is the pain coming from?”, “Will it ever go away?”, or “If this continues, I’ll probably end up in a wheelchair.” Experts refer to these kinds of thoughts as **catastrophizing**. This doesn’t necessarily mean expecting disasters, but rather overthinking the pain, exaggerating its consequences for overall health, and feeling powerless in the face of it.

Why is catastrophizing so problematic? Research suggests that interpreting pain as threatening often leads to fear of movement and injury. For example: “If this pain continues, I’ll probably end up in a wheelchair.” This fear can promote what's known as **avoidance behavior** — deliberately steering clear of activities perceived as potentially harmful. In turn, this avoidance can contribute to the persistence of pain. In contrast, people who don’t engage in catastrophic thinking are less likely to develop pain-related fears. They tend to stay physically active, which can support a quicker recovery from back pain.

So what can be done to counteract catastrophizing tendencies? Therapists often use **attention diversion techniques** — helping individuals learn to focus less on negative thoughts and more on other, often positive, aspects of life. It also involves developing helpful, realistic, and calming thoughts, and building a sense of internal distance from these unhelpful thought patterns.

You can begin practicing these strategies on your own. But if it feels difficult, don’t hesitate to seek professional support. Working through catastrophic thoughts is often much easier — and more effective — when done together.

**Script for the module on Depression**

Many patients with back pain suffer from feelings of sadness, loss of interest, and lack of motivation. Frequently, issues such as sleep problems, changes in appetite, feelings of guilt, or concentration difficulties also arise. These symptoms may be signs of a depressive mood. Patients are often confused about the connection between depression, a mental disorder, and back pain, a physical ailment. However, many studies and investigations have shown that mental disorders are often accompanied by physical symptoms. At the same time, persistent pain is a continuous burden that can also affect the psychological well-being of those affected. What came first, the pain or the emotional disturbance, is often difficult to determine.

Much more important than the question of what is cause and what is effect are the possibilities for changing depressive moods and pain. Often, those affected are caught in so-called vicious circles: they experience pain and feel bad, so they cancel appointments or do not exercise. This, in turn, further lowers their mood, and the back pain intensifies due to the lack of movement. Even more appointments and activities are canceled. The result: they blame themselves for not being able to manage their problems, and the pain continues to worsen.

Breaking free from these patterns becomes increasingly difficult over time. This makes it even more important to seek support if you notice that the problems are increasing, or if you can no longer change your thinking and behavior patterns on your own.

Talking with friends and family, and bringing your general practitioner on board, can be important first steps in helping you feel better again. They can all assist in helping you make your daily life more active, shed negative thought patterns, and rediscover more joy. Therefore, do not wait too long. The sooner you seek help, the faster you can start feeling better. And then your back pain will also affect you much less.

**Script for the module on Fear of Movement**

When it comes to back pain, experts often refer to a vicious cycle of rest and pain. But what exactly does that mean? When we experience pain and rest (meaning we stop engaging in physical and strenuous activities), this helps reduce the pain in the short term. However, if the pain persists and we continue to rest, it leads to a progressive decline in our physical fitness. Muscles weaken, and we have less strength and endurance. This can also lead to tension, poor posture, and a reduction in performance. We start to feel pain more quickly, even with smaller movements. The result? We rest even more because this initially helped, and as a result, our physical fitness continues to decline.

So, how can we prevent this development and break out of the vicious cycle? Through the gradual buildup of physical activity and exercise, fear can be continuously reduced. Initially, simple relaxation or coordination exercises can be helpful. The next step is to deliberately face everyday situations that trigger fear or pain and try to handle them successfully. One starts with "easier" situations and gradually increases the difficulty. This not only reduces our fears, but our body also learns again that appropriate movement can lead to pain relief. In general, physical activity has an antidepressant effect, boosts self-esteem, and reduces stress. And not only that: Studies have shown that exercise activates the body's natural pain inhibition, strengthens the immune system, and prevents many illnesses.

The first step to breaking out of the vicious cycle of rest and back pain is to become active again. This step is often the most difficult. And even if things don't immediately go exactly as we would like - improvements can only be achieved through change.

**Script for the module on Health Anxiety**

Back pain doesn’t just cause physical discomfort — it’s often accompanied by varying degrees of fear. These fears might involve worries about an underlying injury or illness, concerns that the pain will get worse or never go away, or anxiety about no longer being able to do certain activities. The tricky part is that it doesn’t matter whether these fears are actually justified. Our body doesn’t care how realistic a fear is: when we feel afraid, it reacts with typical anxiety symptoms. The nervous system becomes activated, and we might experience a racing heart, trembling, or shortness of breath.

This leads to an additional problem: the activation of the nervous system is not only uncomfortable, but it also causes muscle tension and tightness — which can increase back pain. In fact, fear often makes pain feel worse, and the pain, in turn, fuels the fear.

Once fear takes hold, it often causes us to avoid thoughts or activities that might actually be pleasant or helpful, while focusing even more on what seems threatening. And when we actively search for warning signs, we’re likely to find them — or assign great importance to things that might otherwise go unnoticed. This doesn’t mean we’re imagining the pain; rather, fear reactions have more physical effects on the body than we typically realize.

The muscle tension triggered by fear can intensify back pain. But the good news is that if we can manage our fear, we may also ease some of the pain. There are various ways to positively influence fear related to back pain — including relaxation techniques and breathing exercises. These can help calm the nervous system, redirect our attention away from the pain, and improve sleep and overall recovery. Most importantly, talking about our fears can make a big difference, even if the fears don’t vanish immediately. So if you feel your fears are seriously affecting or limiting you, don’t hesitate to talk to your general practitioner, friends, or family. The less power our fears have over us, the better we’re often able to cope with pain.

**Script for the module on Emotion Regulation**

When it comes to internal states, psychology often distinguishes between bodily sensations and emotions. Although they influence each other, they can be differentiated. For example, we might say, “I feel lethargic, restless, or tense,” but these are actually bodily sensations. Emotions, on the other hand, are feelings like sadness, fear, anger, or joy.

Numerous studies have shown how closely pain and emotions are connected. One example is research finding that the emotional distress people feel when they're excluded from a group activates the same neural networks in the brain as physical pain. Everyday emotional stress—like anger or frustration—can also trigger muscle tension, physical reactions, or even increase pain.

Many people with back pain, in particular, have difficulty distinguishing between their pain and their emotions, especially when it comes to recognizing and expressing how they feel. This can lead to situations where a person experiences physical pain when, in reality, they're feeling anger, disappointment, or worry. When someone struggles to identify their emotions, it also makes it harder to manage them.

The processes we use to consciously influence our emotional experience are known as emotion regulation. In other words, through our thoughts or actions, we can evoke, change, or manage our emotions. If we rely mostly on ineffective coping strategies—like suppressing our feelings or viewing emotions as something negative—we won’t be able to handle them in a healthy, constructive way. And ultimately, that means we won’t be able to positively influence our experience of pain.

A good first step toward positive emotion regulation is becoming more aware of your own emotions. Research has shown that simply labeling how you feel can help reduce unpleasant emotions. One way to build emotional awareness is by keeping a pain-emotion journal. Each time your pain changes, you can write down the situation that triggered it and the emotion you were feeling at the time.

And here’s another tip: when you're experiencing difficult emotions, give them some space. You might vent during a walk or do something that makes you feel good. This can help you better understand your emotions and learn to handle them in a more positive, healthy way.

**Script for the module on Expectations**

A crucial factor in whether our back pain persists, when we’re able to return to work, and how effective a treatment is, lies in our expectations. You’ve probably heard of the so-called placebo effect: when we expect a therapy to help, we’re more likely to feel its positive effects — such as pain relief. But this mechanism also works in the opposite direction. If we expect our symptoms to stay the same or even worsen, that’s often exactly what happens. This negative reinforcement is known as the nocebo effect. Studies have shown, among other things, that negative expectations, worries, or fears can intensify the side effects of a medication.

Our expectations about back pain and its treatment are often shaped by our own past experiences — or the experiences of people around us. Media reports can also amplify negative expectations. The distressing story of a neighbor or a TV show featuring severe cases of back pain can make us fear a poor outcome for ourselves. Expectations like “My back pain will definitely get worse” can trigger signals in our brain and body that sharpen our focus on those symptoms. As a result, the pain takes center stage and is perceived even more intensely. Another problem is that people often hold on tightly to their expectations, even when they’ve been proven wrong or haven’t been confirmed at all.

So how can we let go of these negative fears? An important first step toward approaching back pain with greater optimism is to take a closer look at our expectations. What evidence supports the idea that the pain might worsen — and what speaks against it? What would a realistic expectation look like? Try to actively direct your attention to impressions that support a positive interpretation of the situation, rather than automatically searching for signals that confirm your fears. In fact, you’ve already achieved something meaningful: you chose to watch this video. A part of you already believes that support might be helpful. And now, you can build on those positive expectations.

**Script for the module on Pain Endurance**

Pain researchers believe there are different ways people process pain, and these coping styles can play a role in the development of chronic pain. At one extreme is anxious rest or avoidance behavior; at the other is the tendency to suppress pain sensations.

Pronounced avoidance behavior leads to physical inactivity. When we stop moving, our overall fitness declines, muscles weaken, and processes in the brain are triggered that promote the persistence of back pain — a phenomenon known as pain chronification.

On the flip side, you’ve probably heard sayings like “No pain, no gain.” Many people try to suppress or downplay their back pain. While it can be helpful not to give pain too much importance, this becomes problematic when it leads us to ignore our physical limits. Constantly pushing through severe pain can overstrain the body’s structures, causing repeated micro-injuries in the spine. This also contributes to chronic back pain. People who fall into this pattern often end up exhausted, struggling through their daily routines without lasting improvement.

So how can we find a balance between these two extremes — between rest and overexertion? Balanced coping starts with addressing the reality of our back pain. This includes accepting that our mobility might currently be limited and questioning the expectations we place on ourselves. Why is it so important to do things exactly as we always have? At the same time, it’s important to continue pursuing goals and activities that matter to us.

By learning to recognize our physical limits, we can structure our activities more consciously and gradually increase them over time. For example, we might start with 10 minutes of activity on the first day, then increase to 15 minutes on the second, and continue in small, manageable steps. This gradual approach can help us regain — or even improve — our previous fitness and well-being, because now we’re moving in a structured, thoughtful, and sustainable way.

**Script for the module on Traumatization**

Whether physical or emotional, painful events in our lives can increase our vulnerability to pain disorders. Research has shown that prolonged stress or painful experiences early in life — such as childhood neglect or abuse — can raise the risk of developing back pain later on. Several studies have found that women with traumatic childhood experiences are more likely to suffer from chronic back pain in adulthood compared to those without such experiences. Stressful situations in our current lives can then act as triggers, setting off episodes of back pain.

This connection can be explained by the way stress accumulates in the body over time. When our mind and body are exposed to many negative experiences early on, it can impair our ability to regulate this built-up stress. This affects the immune and hormonal systems, and can lead to disruptions in neural and cognitive functions. As a result, our body becomes less capable of coping with new stress. Additionally, the so-called **pain memory** — unconscious memories of pain stored in the body — can reactivate pain experiences from the past. A current stressor can therefore trigger pain that may seem unrelated from an outside perspective.

When coping with back pain, it may be important to address past traumas. This is best done with professional support. In psychotherapy, people learn strategies to reduce the impact of trauma memories, regain emotional stability, and better manage pain. A central part of therapy involves talking about these experiences — something that can be very difficult at first, but becomes more manageable over time. Your general practitioner can help guide you to the right treatment. By learning to process and come to terms with the past, you’ll be better equipped to handle pain in the present.
